# Electrospun poly(vinyl alcohol)/hyaluronic acid nanofiber mats for wound care in space: cytocompatibility and effects of UV-C treatment and simulated microgravity

**DOI:** 10.3389/fbioe.2026.1946755

**Published:** 2026-10-02

**Authors:** Gianluca Ciarleglio, Alessia Di Pauli, Marika Berardini, Federica Campolo, Angela Catizone, Maria Gabriella Santonicola

**Affiliations:** 1 Department of Chemical Engineering Materials Environment, Sapienza University of Rome, Rome, Italy; 2 Department of Anatomy, Forensic-Medicine and Orthopaedics, Sapienza University of Rome, Rome, Italy; 3 Unicamillus-Saint Camillus International, University of Health Sciences, Rome, Italy

**Keywords:** cytocompatibility, electrospun nanofibers, hyaluronic acid, simulated microgravity, UV-C sterilization, wound dressing

## Abstract

Electrospun nanofibrous dressings are attractive for wound-care applications in space-related healthcare; however, limited information is available on the stability of UV-C-treated, water-stabilized nanofiber mats after exposure to simulated microgravity and on their cytocompatibility after pre-use treatment. To address this gap, fully aqueous poly(vinyl alcohol) (PVA) and poly (vinyl alcohol)/hyaluronic acid (PVA/HA) electrospun mats were stabilized through citric acid-mediated ester crosslinking and evaluated after UV-C treatment followed by 48 h incubation under static or simulated microgravity conditions. Both x-PVA and x-PVA/HA retained a continuous fibrous architecture without network collapse, although simulated microgravity was associated with formulation-dependent morphological remodeling. x-PVA showed a more pronounced increase in fiber diameter and a coarser network, whereas x-PVA/HA exhibited a more moderate diameter increase while retaining a highly interconnected fibrous organization. Water vapor transmission rates of 2,552.6 ± 66.7 and 2,492.6 ± 21.6 g m^−2^ day^−1^ were measured for x-PVA and x-PVA/HA, respectively, indicating substantial vapor transport under the investigated conditions. FTIR-ATR analysis showed preservation of the characteristic bands of the ester-crosslinked PVA-based networks, without evidence of marked chemical degradation. DSC revealed treatment-dependent changes in thermal transitions, whereas simulated microgravity produced no pronounced additional shift compared with the corresponding static controls. *In vitro* assays performed on UV-C-treated x-PVA/HA supported cytocompatibility across the tested cell models, including human keratinocytes. Overall, citric-acid-crosslinked x-PVA/HA nanofibrous mats combined breathable vapor-regulating behavior with preserved fibrous architecture and physicochemical stability after UV-C treatment and short-term simulated microgravity exposure. Together with the observed *in vitro* cytocompatibility, these findings support their further investigation as wound-contact materials for space-related healthcare.

## 1 Introduction

Wound management is a relevant requirement in both terrestrial healthcare and space medicine (Sen et al., 2009; Jemison and Olabisi, 2021). In conventional clinical practice, wound dressings are designed to protect the wound bed, regulate moisture and vapor exchange, limit microbial contamination, and support tissue repair (Dhivya et al., 2015). These requirements become more demanding during long-duration exploration missions, where medical operations must become progressively more Earth-independent because of limited resupply, restricted mass and storage volume, communication latency, limited evacuation opportunities, and the need for greater onboard medical autonomy (Levin et al., 2023; Anderson et al., 2025). In this context, wound dressings should not only be cytocompatible and functionally effective, but also able to retain their properties after pre-use processing, transport, and storage (Jemison and Olabisi, 2021).

Skin health and wound repair are increasingly recognized as relevant aspects of human spaceflight. Spaceflight has been associated with skin dryness, irritation, sensitivity, thinning, infections, abrasions, delayed wound healing, and accelerated skin aging (Farkas and Farkas, 2021). Recent molecular evidence further indicates that even short-duration spaceflight can induce responses associated with inflammation, epithelial barrier disruption, DNA damage, and impaired regeneration (Park et al., 2024). In addition, microgravity, radiation, immune dysregulation, microbiome changes, and altered mechanical loading may collectively affect skin homeostasis and repair (Radstake et al., 2022). Ground-based simulated microgravity studies have reported impaired fibroblast migration, changes in cytoskeletal organization, and altered communication among keratinocytes, fibroblasts, and endothelial cells (Zhou et al., 2023; Radstake et al., 2023; Zhao et al., 2025). These findings support the need for wound dressing materials whose structure and biological response remain reliable under altered-gravity-related storage or exposure conditions.

Electrospun polymeric nanofibers are particularly attractive for wound dressing applications because their architecture reproduces several features of the extracellular matrix, including high surface area, interconnected porosity, and a fibrous morphology favorable for vapor exchange and tissue-interface interactions. Electrospun membranes can support moisture regulation, gas exchange, bioactive molecule incorporation, and cell–material interactions, making them suitable for advanced wound care (Yang and Xu, 2023; Chen et al., 2022; Zhou et al., 2025). Electrospun wound dressing systems have been reported with hierarchical architectures for antibacterial activity and cytocompatibility (Xu et al., 2022), PVA-based mats incorporating bioactive glass, silver nanoparticles, and curcumin for *in vitro* and *in vivo* wound healing (Mahmoudi et al., 2023), and piezoelectric nanocomposite electrospun dressings designed to modulate scar-related repair processes (Zhang et al., 2025). These studies confirm the strong interest in electrospun biomaterials that integrate structural design, biological response, and wound-care performance.

Among water-processable polymers, poly (vinyl alcohol) (PVA) is widely used for electrospun biomedical membranes because of its hydrophilicity, biocompatibility, film-forming ability, and processability in aqueous media. However, the high-water solubility of PVA remains a major limitation for applications involving contact with biological fluids. Chemical stabilization is therefore required to preserve membrane integrity under physiological-like conditions. Citric acid (CA) offers a biocompatible route to stabilize PVA-based networks through thermally induced esterification between CA carboxyl groups and PVA hydroxyl groups, avoiding more aggressive or potentially toxic crosslinking agents (Nataraj et al., 2020; Ciarleglio et al., 2026a; Ciarleglio et al., 2026b). Hyaluronic acid (HA) is also highly relevant for wound dressing design because it is a naturally occurring extracellular matrix polysaccharide involved in hydration, cell migration, tissue remodeling, and repair. HA-containing electrospun systems have been investigated as bioactive wound dressings, including PVA/HA-based nanofibers loaded with L-arginine (Hussein et al., 2020), while HA-based biomaterials have been explored for their ability to modulate cell–material interactions and mechanobiological responses (Klimovič et al., 2024).

For wound-contact biomaterials, pre-use decontamination or sterilization steps are necessary before biological testing and intended application. UV-C irradiation is an attractive non-thermal surface treatment for polymeric materials and temperature-sensitive systems; nevertheless, UV-based treatments may alter polymer chemistry, surface properties, chain organization, mechanical response, thermal behavior, and cell–material interactions (Fischbach et al., 2001; Kanjickal et al., 2008; Chansoria et al., 2020). Accordingly, the effect of UV-C exposure should be assessed directly on the specific material architecture under investigation.

For wound dressings intended for space-related healthcare, these aspects become particularly relevant because pre-sterilized materials may need to retain their properties after pre-use processing and storage under non-standard environmental conditions. Despite the increasing interest in electrospun wound dressings and biomaterials for space medicine, limited information is available on the behavior of UV-C-treated, ester-crosslinked PVA-based nanofibrous mats after short-term exposure to simulated microgravity. This represents an important knowledge gap for wound dressing systems intended for non-standard healthcare environments, including space-related scenarios, where material stability after sterilization, transport, and storage is essential before use.

In this work, we investigate the response of citric-acid-crosslinked x-PVA and x-PVA/HA electrospun nanofiber mats to UV-C treatment followed by short-term incubation under static or simulated microgravity conditions. The results show that both formulations retain their fibrous architecture after treatment, with formulation-dependent morphological remodeling under simulated microgravity, while maintaining substantial water vapor transmission and the main physicochemical features of the ester-crosslinked networks. *In vitro* assays on UV-C-treated x-PVA/HA further support its cytocompatibility under the investigated conditions. These findings provide a preliminary assessment of x-PVA/HA nanofibrous membranes as wound-contact materials requiring stability after pre-use treatment and short-term exposure to simulated microgravity-related conditions.

## 2 Experimental section

### 2.1 Materials

Poly(vinyl alcohol) (PVA, Mw 130 kDa, 86.7%–88.7% hydrolyzed), anhydrous citric acid (CA), and phosphate-buffered saline (PBS, P4417; 0.01 M phosphate buffer, 0.0027 M KCl, 0.137 M NaCl) were purchased from Merck Life Science (Milan, Italy). Sodium hyaluronate, hereafter referred to as hyaluronic acid (HA, Mw 1,500–1,800 kDa), was obtained from ACEF (Piacenza, Italy). Milli-Q ultrapure water (resistivity 18.2 MΩ cm, Milli-Q system, Merck Millipore, Burlington, MA, United States) was used for the preparation of all aqueous solutions. Unless otherwise stated, all reagents were used as received.

NT2D1 human embryonal carcinoma cells, HEK293 human embryonic kidney cells, and HaCaT human keratinocytes were obtained from the American Type Culture Collection (ATCC, Manassas, VA, United States). Dulbecco’s modified Eagle’s medium (Sigma Aldrich, cat. D6546, St. Louis, MO, United States), L-glutamine (Sigma Aldrich, cat. G7513, St. Louis, MO, United States), penicillin/streptomycin (Sigma-Aldrich, cat. P0781, St. Louis, MO, United States), propidium iodide (PI, Sigma-Aldrich, cat. P4864, St. Louis, MO, United States), and the PKH67 Green Fluorescent Cell Linker Kit (PKH67GL-1KT) were purchased from Merck Life Science (St. Louis, MO, United States). Fetal bovine serum (FBS, 10270), trypsin-EDTA (0.25%, 25200056), Opti-MEM (31985-047), Lipofectamine 3000 reagent (L3000013), and P3000 reagent were obtained from Thermo Fisher Scientific/Gibco (Grand Island, NY, United States). μ-Slide 8-well chambers were purchased from Ibidi GmbH (80807, Martinsried, Germany). Paraformaldehyde was obtained from Electron Microscopy Sciences (Hatfield, PA, United States), while rhodamine-phalloidin was purchased from Invitrogen Molecular Probes (Eugene, OR, United States). The peGFP-C1 plasmid and buffered glycerol mounting medium were used for GFP transfection and fluorescence microscopy, respectively.

### 2.2 Fabrication of electrospun nanofiber mats

x-PVA and x-PVA/HA nanofiber mats were fabricated by electrospinning using the aqueous formulations and processing parameters previously optimized for the fabrication of chemically stabilized PVA-based nanofibrous membranes (Ciarleglio et al., 2026a). Briefly, PVA was dissolved in ultrapure water at 80 °C under magnetic stirring for 1 h. For HA-containing formulations, HA was subsequently added at 50 °C and the solution was stirred for an additional 1 h. After cooling to room temperature, CA was added and the final solution was stirred for 30 min before electrospinning. Based on the previously optimized composition, the final formulations contained 7 wt% PVA, 0.25 wt% CA, and, when present, 0.25 wt% HA.

In the present study, the optimized process was transferred from a single-needle configuration to a multi-needle electrospinning setup equipped with four stainless-steel needles. Each needle had an inner diameter of 0.514 mm (21 G), and the polymer solution was delivered at 0.5 mL/h per needle, for a total flow rate of 2.0 mL/h. The applied voltage was set at 20 kV, and the collecting distance was maintained at 10 cm. Nanofibers were collected on aluminum foil under controlled environmental conditions, maintaining the temperature at approximately 25 °C and the relative humidity below 50%. A custom-made experimental setup for electrospinning/electrospraying was used to fabricate the nanofibers (Ciarleglio et al., 2025; Ciarleglio et al., 2025a; Ciarleglio et al., 2024). After electrospinning, the nanofiber mats were thermally treated at 150 °C for 2 h to promote solid-state esterification between the hydroxyl groups of PVA/HA and the carboxyl groups of CA. This treatment resulted in ester-crosslinked and water-stabilized x-PVA and x-PVA/HA nanofibrous networks.

### 2.3 UV-C treatment and simulated microgravity exposure

After thermal stabilization, x-PVA and x-PVA/HA nanofiber mats underwent post-fabrication treatments to evaluate the effects of UV-C sterilization and simulated microgravity exposure on their morphology and physicochemical stability. For each formulation, three experimental conditions were considered: untreated nanofibers, UV-C-sterilized nanofibers maintained under static conditions at 37 °C, and UV-C-treated nanofibers exposed to simulated microgravity at 37 °C.

The UV-C treatment was performed using a UV-Sterilizer YM-9003 lamp (5 W, DM Soluzioni ICT, Pescara, Italy) emitting at 254 nm. Nanofiber mats were placed at a distance of 15 cm from the UV-C source, corresponding to an irradiance of 17.70 W m^−2^. Irradiation was carried out for 1 h, resulting in a total UV-C dose of 6.37 × 10^4^ J m^−2^ (Blondelli et al., 2026). After UV-C irradiation, samples were incubated for 48 h under either static conditions or simulated microgravity. Both static and simulated microgravity-exposed samples were maintained in the same humidified incubator at 37 °C with 5% CO_2_. Static control samples were placed in the same incubator and maintained under identical temperature, humidity and CO_2_ conditions as the RPM-exposed samples, allowing RPM-based continuous reorientation to be compared with matched static incubation. Simulated microgravity exposure was performed using a desktop random positioning machine (RPM; Dutch Space, Leiden, Netherlands) connected to a control console. The RPM consisted of two independently rotating frames that continuously reoriented the samples during exposure, according to a ground-based simulated microgravity approach. The resulting sample groups were named x-PVA, x-PVA UV-C (37 °C), x-PVA UV-C + μg (37 °C), x-PVA/HA, x-PVA/HA UV-C (37 °C), and x-PVA/HA UV-C + μg (37 °C). A representative schematic illustration of the experimental workflow and of the positioning of the nanofiber mats during simulated microgravity exposure using the RPM is shown in Figure 1.

**FIGURE 1:**
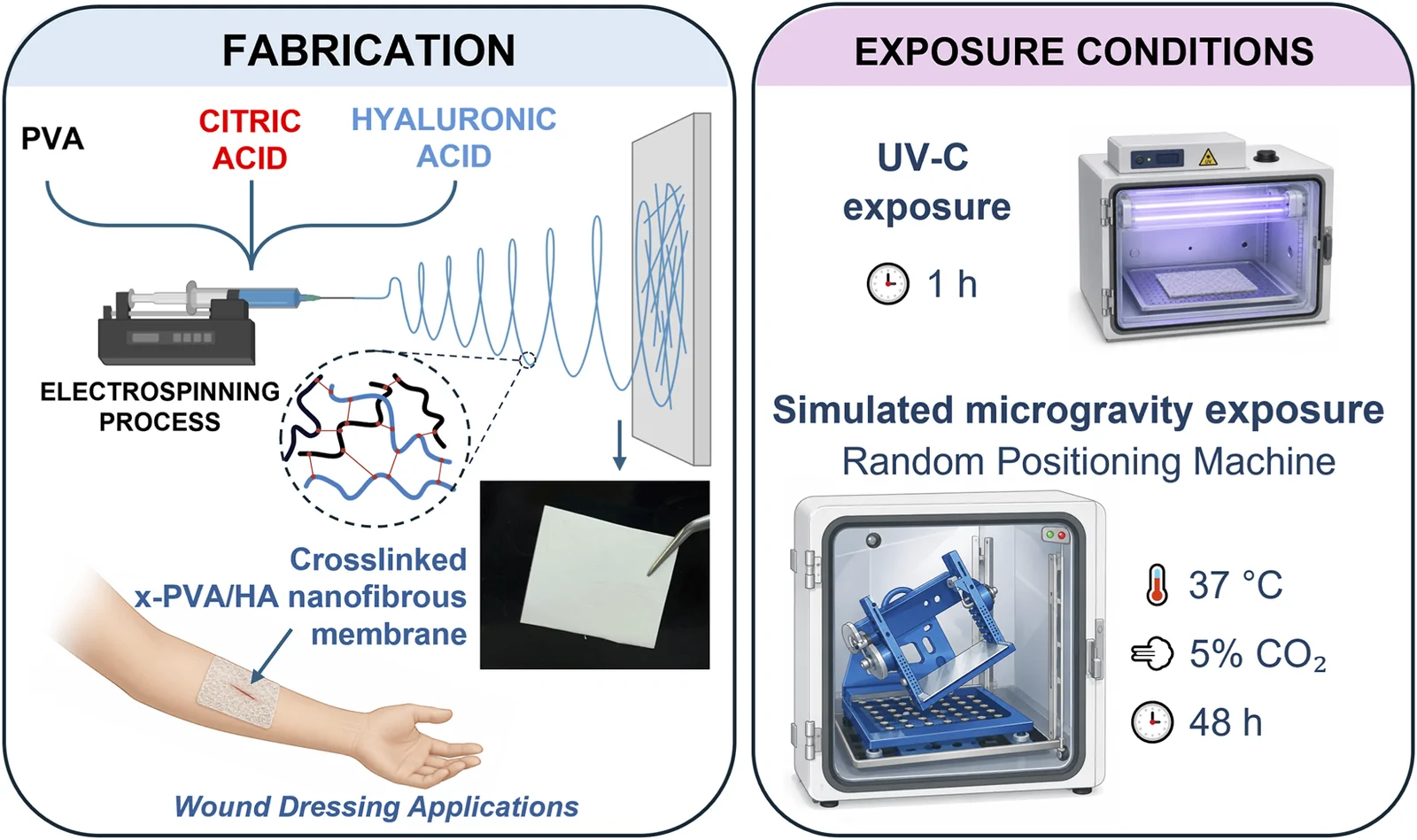
Schematic representation of the experimental workflow used to evaluate the effects of UV-C treatment and simulated microgravity on electrospun x-PVA and x-PVA/HA nanofiber mats. After UV-C irradiation, the samples were incubated for 48 h under static conditions or exposed to simulated microgravity using a random positioning machine (RPM) at 37 °C and 5% CO_2_.

### 2.4 Characterization methods

#### 2.4.1 Morphological characterization

The morphology and microstructural features of the nanofibers were examined by scanning electron microscopy (SEM) using a VEGA II LSH instrument (Tescan, Brno, Czech Republic). Images were acquired at an accelerating voltage of 20 kV at magnifications of 20,000× and 40,000×. Before imaging, the nanofibers were coated with gold using a Cressington 108 auto sputter coater (Cressington Scientific Instruments, Watford, United Kingdom). Fiber diameter measurements were carried out using a modified version of the SIMPoly algorithm implemented in MATLAB R2024b (MathWorks, Natick, MA, United States) (Pini et al., 2026). For each experimental condition, fiber diameter distributions were obtained from n = 100 fiber measurements extracted from representative SEM micrographs. The resulting values are reported as mean ± standard deviation. The effects of membrane composition and post-treatment condition on fiber diameter were evaluated by two-way analysis of variance (ANOVA), including the composition × treatment interaction. Statistical analysis was performed using MATLAB R2024b (MathWorks, Natick, MA, United States). In addition to fiber diameter analysis, descriptive network descriptors were extracted from the representative 40,000 × SEM micrographs using the same MATLAB-based image-processing workflow. After image calibration, segmentation, and binarization, void regions were identified from the background phase of the binary images. The mean void equivalent diameter was calculated as the diameter of a circle having the same area as each detected void. Void density was calculated as the number of voids normalized to the analyzed image area and expressed as voids/100 μm^2^. Junction density was calculated after skeletonization of the fibrous network by identifying fiber intersection points and normalizing their number to the analyzed image area; values were expressed as junctions/100 μm^2^. These network descriptors were used as semi-quantitative parameters to support the comparison of representative SEM images and to describe treatment-induced network remodeling. For each experimental condition, network descriptors were calculated independently within three non-overlapping regions of interest (ROIs) of the representative 40,000 × SEM micrograph. Results are reported as mean ± SD from three ROIs per condition (n = 3 ROIs).

#### 2.4.2 Water vapor transmission rate (WVTR)

The water vapor transmission rate (WVTR) of the electrospun nanofiber mats was determined using a gravimetric cup method adapted from ASTM E96 and EN ISO 7783 (Ciarleglio et al., 2025b). x-PVA and x-PVA/HA membranes were fixed onto the opening of a cylindrical container containing 20 mL of PBS at pH 8, selected to mimic the alkaline microenvironment of a wound. The internal diameter of the exposed area was 2.3 cm, corresponding to an exposed membrane area of 4.15 cm^2^ (4.15 × 10^−4^ m^2^). The volume of PBS was adjusted to maintain a distance of ∼2.0 cm between the liquid surface and the membrane. The assembled systems were sealed to ensure that water vapor exchange occurred only through the exposed membrane area and incubated at 37 °C. The mass of each system was recorded at 1 h intervals until a steady-state regime was reached. For each sample, the linear region of the mass loss curve was fitted by least-squares linear regression, and the slope was used to calculate the WVTR according to Equation 1:

$$WVTR=\left(\frac{G/t}{A}\right)$$ (1)

where G/t is the slope of the mass loss curve (g h^−1^), and A is the exposed membrane area (m^2^). The goodness of the linear fit was evaluated using the coefficient of determination (*R* ^2^). Measurements were performed on three independent replicates for each membrane formulation and for the blank control (n = 3 per condition). Blank cups without membranes were included to monitor free PBS evaporation under the same experimental conditions and were used as experimental controls.

#### 2.4.3 FTIR-ATR spectroscopy

Fourier-transform infrared spectroscopy in attenuated total reflectance mode (FTIR-ATR) was used to investigate the chemical structure of the nanofiber mats and to evaluate possible chemical modifications induced by UV-C sterilization and simulated microgravity exposure. Spectra were acquired using a Nicolet Summit FTIR spectrometer (Thermo Fisher Scientific, Waltham, MA, United States) equipped with a diamond ATR crystal. Each spectrum was collected in the wavenumber range of 500–4,000 cm^−1^, with 64 scans and a spectral resolution of 4 cm^−1^. Baseline-corrected spectra were used for comparative analysis. The peak-height ratio between the C–H stretching bands at 2,941 and 2,915 cm^−1^ was calculated as a semi-quantitative descriptor of relative spectral changes within the C–H stretching envelope. This ratio was used only to support comparison among treatment conditions and was not intended as an absolute quantitative measure.

#### 2.4.4 Thermal characterization

Thermal analysis was performed by differential scanning calorimetry using a DSC 8500 instrument (PerkinElmer, Waltham, MA, United States) calibrated with high-purity indium and tin. Experiments were carried out under a nitrogen flow of 40 mL/min on samples of ∼10 mg sealed in aluminum pans with lids. Thermograms were acquired in the temperature range from 25 °C to 250 °C at a heating rate of 10 °C/min. DSC analysis was performed on x-PVA and x-PVA/HA nanofiber mats under the different experimental conditions: untreated, UV-C-sterilized and incubated at 37 °C for 48 h under static conditions, and UV-C-sterilized and exposed to simulated microgravity at 37 °C for 48 h. The glass transition temperature (T_g_), melting temperature (T_m_), heat capacity change associated with the glass transition (ΔC_p_), and melting enthalpy (ΔH_m_) were determined from the thermograms. DSC measurements were performed on three independent specimens per experimental condition (n = 3).

#### 2.4.5 Density measurement

The density of the thermally stabilized x-PVA and x-PVA/HA nanofiber mats was measured by hydrostatic weighing in ultrapure water, following ASTM D792 guidelines. Measurements were performed using a Sartorius YDK01 density determination kit installed on a CPA225D analytical balance (Sartorius, Göttingen, Germany). Samples were weighed both in air and immersed in ultrapure water, and density values were calculated by taking into account the density of the immersion liquid, air buoyancy, and the correction factor of the measurement kit. Five independent specimens were analyzed for each formulation, and results are reported as mean ± standard deviation.

### 2.5 Cell culture and cytocompatibility assays

#### 2.5.1 Cell culture

NT2D1 human embryonal carcinoma cell line, HEK293 human embryonic kidney cell line and HaCaT human keratinocyte cell line were seeded at 5 × 10^4^ cells/cm^2^ on the sterilized x-PVA/HA membrane placed in μ-Slide 8-well chambers and cultured in DMEM complemented with 10% fetal bovine serum, 1% L-glutamine and 2% penicillin/streptomycin at 37 °C in a humidified atmosphere with 5% CO_2_ for different time. Control cells were cultured at the same time without x-PVA/HA. Finally, the x-PVA/HA was removed and observed with optical microscope, while the cells were processed as described below.

#### 2.5.2 Actin visualization

Actin microfilaments were analyzed in NT2D1 cells adherent to the substrate. NT2D1 adherent cells were fixed with 4% paraformaldehyde (cat. n.157-8 Electron Microscopy Sciences) for 20 min at 4 °C and washed twice for 10 min with Phosphate-Buffered Saline (SIAL-PBS-2a); for F-actin visualization, rhodamine phalloidin (Invitrogen Molecular Probes, Eugene, OR, United States; 1:50 dilution) was used. Cells were then washed in PBS and mounted in buffered glycerol (0.1 M, pH 9.5). Finally, cells were observed with Zeiss confocal microscopy LM900 (Zeiss, Oberkochen, Germany; Ex-Em 550-573 nm) and images were captured using a 20× Objective.

#### 2.5.3 Viability by Adhesion assay

Adhesion assay was performed on NT2D1 cells. Following the 24 h direct contact exposure with x-PVA/HA (as described above), NT2D1 cells were detached using 0.25% trypsin-EDTA (Gibco, cat. 25200056, Grand Island, NY, United States) for 5 min at 37 °C. Trypsin was neutralized with complete DMEM medium, and cells were centrifuged at 400 *g* for 5 min. The pellet was resuspended, and cells were reseeded in μ-Slide 8-well chambers to assess viability by plastic adhesion recovery. Cultures were maintained for 72 h at 37 °C, 5% CO_2_ and captured by an inverted phase-contrast microscope (Nikon Eclipse, TS100) using a 40× Obj.

#### 2.5.4 GFP transfection

GFP-Transfection was performed on HEK293 cells. The cells were seeded at 2 × 10^5^ cells per 35 mm Petri dish in 2.5 mL complete DMEM and incubated overnight at 37 °C, 5% CO_2_ to reach 70%–80% confluence. After 24 h, the medium was replaced with fresh complete DMEM, and cells were transfected with the pEGFP-C1 plasmid (2.5 μg/μL) using Lipofectamine 3000 as follows: mix 1: 120 μL Opti-MEM (Gibco, cat. 31985-047) + 5 μL Lipofectamine 3000 Reagent; mix 2: 2 μL pEGFP-C1 plasmid (5 μg total) + 10 μL P3000 Reagent +13 μL Opti-MEM. Both mixes were incubated separately for 15 min at RT, then combined and added dropwise to the dish. Transfected cultures were maintained for 24 h at 37 °C, 5% CO_2_. Transfection efficiency was assessed by detecting GFP fluorescence by Nikon Eclipse Ts2.

#### 2.5.5 PKH67 labelling

PKH67 Green Fluorescent Cell Linker Kit labelling was performed on HaCaT cells. Briefly, cell suspension (5 × 10^5^ cells) in 200 µL PBS was mixed in equal amounts of solution consisting of 2 µL of PKH67 in 200 µL of Diluent C and incubated for 5 min at RT in darkness. Then the cells were washed three times with serum/media, centrifuged at 400 *g* for 5 min and seeded in serum/media in μ-Slide 8-well chambers. Green fluorescence was analysed by Zeiss LM900 confocal microscope (20× objective, PKH67 ex 488 nm). Optical spatial series with step size of 1µm were performed. For HaCaT cells, cell death was quantified from the confocal microscopy series as the ratio between the sum intensity of PI-positive red fluorescence and the sum intensity of PKH67-positive green fluorescence.

#### 2.5.6 Viability by PI exclusion assay

Transfected GFP-HEK293 and PKH67-labeled HaCaT cells were seeded at 5 × 10^4^ cells per well in μ-Slide 8-well chambers and exposed to the sterilized x-PVA/HA for 24 h and 48 h, respectively, as described in the cell culture section. Cells were treated with propidium iodide (PI) to perform live cell imaging of both HEK293 cells and HaCaT cells as follows: 2 μg/mL of PI solution was added to each sample for 5 min, and PI fluorescence was determined by Zeiss LM900 confocal microscope (×20 objective, GFP ex 488 nm, PKH67 ex 488 nm, PI ex 561 nm). Total (green fluorescence) and dead (red fluorescence) cells were analysed. Optical spatial series with a step size of 1 µm were performed.

### 2.6 Statistical analysis

Quantitative data are presented as mean ± standard deviation (SD) or mean ± standard error of the mean (SEM), as specified for each analysis. Fiber-diameter distributions were obtained from 100 individual measurements per experimental condition. Water vapor transmission rate measurements and DSC analyses were performed on three independent specimens per experimental condition (n = 3), while density measurements were performed on five independent specimens per formulation (n = 5). Statistical analyses of material-related quantitative data were performed using MATLAB R2024b (MathWorks, Natick, MA, United States). Depending on the experimental design, one- or two-way analysis of variance (ANOVA) followed by Tukey’s multiple-comparison test was used, as specified in the corresponding figure legends. Cell-based quantitative data are reported as mean ± SEM and were analyzed using Student’s t-test. Statistical significance was set at p < 0.05; ns indicates no statistically significant difference.

## 3 Results and discussion

### 3.1 Morphological analysis of untreated and post-treated nanofiber mats

SEM analysis was performed to evaluate the effect of UV-C treatment and subsequent incubation under either static or simulated microgravity conditions on the morphology of x-PVA and x-PVA/HA nanofiber mats. Representative SEM micrographs and corresponding fiber-diameter distributions are shown in Figures 2a–f, while the quantitative morphological parameters are reported in Figures 2g–j. All samples retained a recognizable fibrous architecture after post-fabrication treatments. UV-C irradiation followed by 48 h incubation at 37 °C, under either static or simulated microgravity conditions, did not induce network collapse or transition to a continuous film-like morphology. However, compared with the UV-C-treated static controls, samples exposed to the UV-C + μg condition showed a more evident rearrangement of the fibrous network, suggesting a formulation-dependent morphological response to the investigated post-treatment conditions.

**FIGURE 2:**
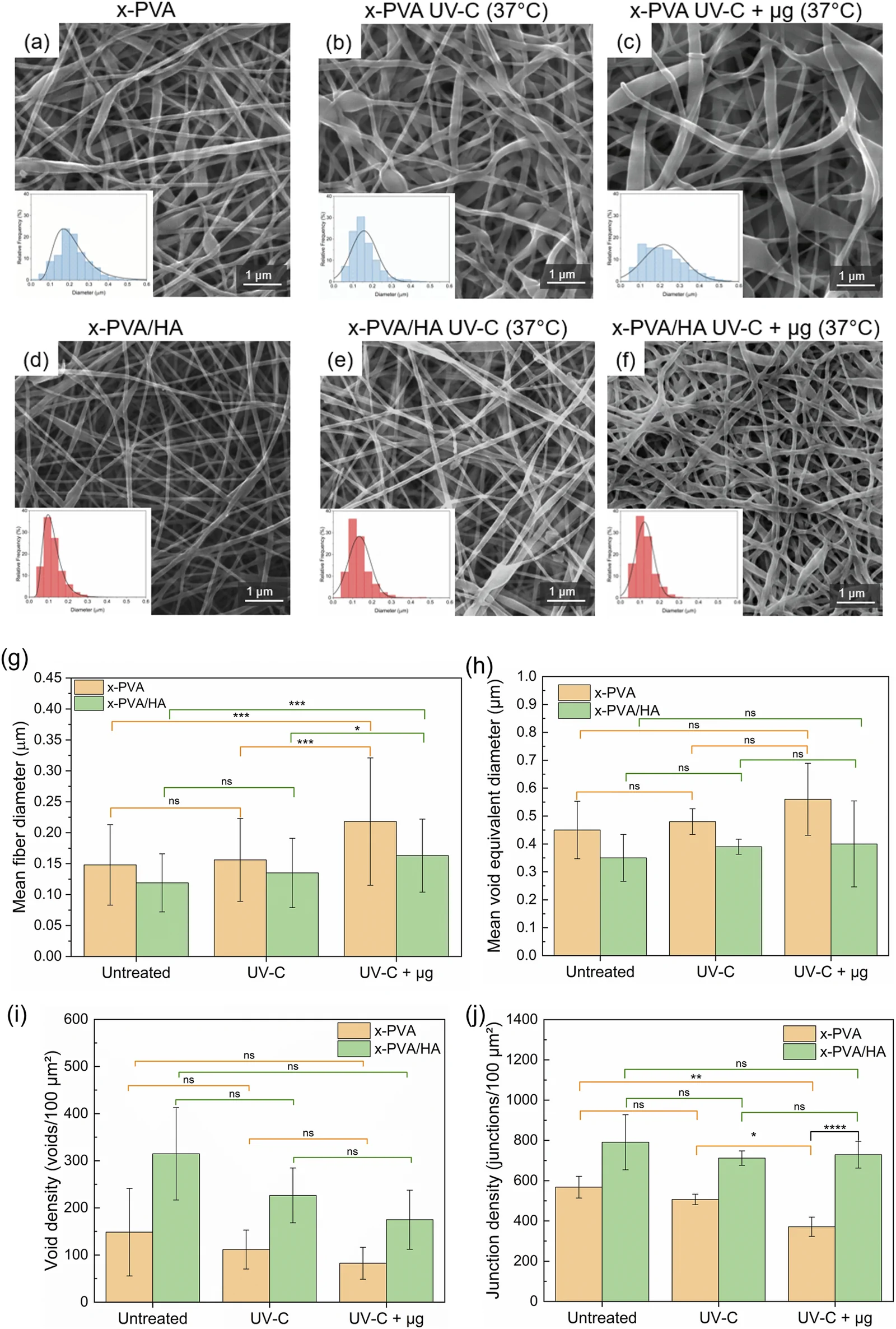
Morphological characterization of untreated and post-treated electrospun x-PVA and x-PVA/HA nanofiber mats. Representative SEM micrographs acquired at 40,000× magnification and corresponding fiber-diameter distributions of **(a)** x-PVA, **(b)** x-PVA UV-C (37 °C), **(c)** x-PVA UV-C + μg (37 °C), **(d)** x-PVA/HA, **(e)** x-PVA/HA UV-C (37 °C), and **(f)** x-PVA/HA UV-C + μg (37 °C). UV-C-treated samples were incubated for 48 h at 37 °C under either static conditions or simulated microgravity. Insets show the corresponding fiber-diameter distributions. Scale bars = 1 µm. Quantitative morphological analysis showing **(g)** mean fiber diameter, **(h)** mean void equivalent diameter, **(i)** void density, and **(j)** junction density. Fiber-diameter data in panel **(g)** are presented as mean ± SD from 100 individual fiber-diameter measurements per experimental condition. Parameters reported in panels **(h–j)** are presented as mean ± SD from three non-overlapping regions of interest (ROIs) analyzed within the representative 40,000 × SEM micrograph for each experimental condition (n = 3 ROIs). Statistical differences were evaluated by two-way ANOVA considering membrane composition and post-treatment condition, followed by Tukey’s multiple-comparison test. Statistical annotations indicate the pairwise comparisons shown in the corresponding panels. *p < 0.05, **p < 0.01, ***p < 0.001, ****p < 0.0001; ns, not significant.

For x-PVA membranes, the untreated sample showed a mean fiber diameter of 0.148 ± 0.065 μm. After UV-C sterilization followed by static incubation at 37 °C, the mean diameter slightly increased to 0.156 ± 0.067 μm. A more pronounced increase was observed after UV-C treatment followed by simulated microgravity exposure at 37 °C, with the mean fiber diameter reaching 0.218 ± 0.103 μm. This corresponds to an increase of ∼47% compared with the untreated membrane and ∼40% compared with the UV-C-treated static control. This trend was consistent with the SEM micrographs, where the x-PVA UV-C + μg (37 °C) sample displayed thicker fibers, larger interfiber voids, and a coarser fibrous network compared with the UV-C-treated static control (Figures 2a–c).

A similar, although less pronounced, trend was observed for x-PVA/HA membranes. The mean fiber diameter increased from 0.119 ± 0.047 μm in the untreated sample to 0.135 ± 0.056 μm after UV-C treatment and static incubation at 37 °C, and to 0.163 ± 0.059 μm after UV-C treatment followed by simulated microgravity exposure at 37 °C. This corresponds to an increase of ∼37% compared with the untreated membrane and ∼21% compared with the UV-C-treated static. SEM images showed that the x-PVA/HA fibrous network was preserved after both treatments. However, the UV-C + μg (37 °C) condition displayed a denser and locally rearranged morphology, with thicker fibers, increased fiber–fiber contacts, and a more continuous appearance along the fiber axis compared with the corresponding untreated and UV-C-treated samples (Figures 2d–f).

Quantitative SEM image analysis further supported the formulation-dependent morphological response (Figures 2g–j). For x-PVA, the mean fiber diameter increased from 0.148 ± 0.065 µm in the untreated membrane to 0.218 ± 0.103 µm after UV-C + μg exposure (Figure 2g). Mean void equivalent diameter and void density showed numerical changes that were not statistically significant, whereas junction density decreased significantly from 568 ± 54 to 371 ± 48 junctions/100 μm^2^ (Figures 2h–j), consistent with reduced network interconnectivity. For x-PVA/HA, the mean fiber diameter also increased significantly, from 0.119 ± 0.047 to 0.163 ± 0.059 µm (Figure 2g), while within-formulation changes in void size, void density, and junction density were not statistically significant. Notably, after UV-C + μg exposure, junction density remained significantly higher in x-PVA/HA than in x-PVA, supporting a greater retention of network interconnectivity in the HA-containing formulation. Overall, these findings support a formulation-dependent rearrangement of the fibrous network under the investigated conditions. The measured fiber diameters fall within the submicrometric range reported for electrospun PVA-based and PVA/HA-based wound dressing materials. Since electrospun fiber morphology is strongly influenced by solution properties and processing conditions (Xue et al., 2019; Baji et al., 2010), the retention of continuous fibrous architectures after treatment indicates that the ester-crosslinked x-PVA and x-PVA/HA membranes maintained their structural organization after UV-C sterilization and short-term incubation under static or simulated microgravity conditions. This behavior is consistent with the material-dependent response of electrospun membranes to sterilization, since sterilization effects are known to depend on polymer chemistry, scaffold architecture, and sterilization method (Rediguieri et al., 2016). Previous studies reported that UV and γ-radiation sterilization can be less detrimental to electrospun PLA fiber morphology than ethylene oxide treatment (Valente et al., 2016), and that UV sterilization combined with short-term storage does not necessarily induce macroscopic collapse of electrospun scaffolds (Evrova et al., 2019). In the present x-PVA and x-PVA/HA membranes, the absence of network collapse indicates that citric-acid-mediated ester crosslinking provided sufficient structural stability to withstand UV-C exposure and subsequent 48 h incubation. The main morphological effect was associated with the UV-C + simulated microgravity condition. This effect was observed in both formulations, but with different morphological signatures. In x-PVA, remodeling mainly appeared as fiber thickening, increased void size, reduced junction density, and network coarsening. In x-PVA/HA, the diameter increase was more moderate, while the network showed local compaction, increased interfiber contacts, and fiber-axis rearrangement.

From a physical standpoint, the observed morphological remodeling may be related to the relaxation of non-equilibrium polymer-chain conformations generated during electrospinning. Rapid jet stretching and solvent evaporation can kinetically constrain polymer chains within electrospun fibers, while subsequent environmental or thermal exposure may promote chain relaxation and structural reorganization (Xue et al., 2019; Baji et al., 2010; Zhang et al., 2022). In hydrophilic PVA-based systems, absorbed water can act as a plasticizer by modifying intermolecular hydrogen-bonding interactions and increasing polymer-chain mobility (Li et al., 2021). Relative humidity is also known to influence the structural organization and morphology of electrospun polymer fibers (Szewczyk and Stachewicz, 2020). Under the humidified incubation conditions used in the present study, increased chain mobility may therefore have contributed to local rearrangement of the fibrous network. Since static and RPM-exposed samples were maintained under identical temperature and humidity conditions, the additional morphological changes observed after RPM exposure were associated with the different mechanical environment generated by continuous sample reorientation. Random positioning machines simulate reduced-gravity conditions through continuous reorientation of the gravity vector, although rotation-related effects and residual accelerations may also contribute to the experimental environment (Wadhwa et al., 2026). Therefore, the present results do not allow the observed remodeling to be attributed to a single mechanical mechanism. Rather, the different responses of x-PVA and x-PVA/HA support a formulation-dependent rearrangement of the fibrous network, with the HA-containing formulation retaining a higher degree of network interconnectivity under the investigated conditions.

As an additional physical descriptor, the apparent density of the thermally stabilized nanofiber mats was measured by hydrostatic weighing. x-PVA mats showed an apparent density of 1.297 ± 0.163 g/cm^3^, while x-PVA/HA mats showed a slightly lower value of 1.194 ± 0.122 g/cm^3^. The lower apparent density of the HA-containing formulation is consistent with differences in fiber packing and network organization, in agreement with the more moderate morphological response observed by SEM.

Two-way ANOVA showed significant effects of membrane composition (p = 7.46 × 10^−10^) and post-treatment condition (p = 1.90 × 10^−16^) on fiber diameter, together with a significant composition × treatment interaction (p = 0.0351). Tukey’s multiple-comparison test further identified treatment-dependent differences within each formulation, as indicated in Figure 2g. Overall, the SEM image analysis showed that UV-C treatment followed by simulated microgravity exposure was associated with measurable morphological remodeling compared with UV-C-treated static controls. x-PVA showed a more pronounced increase in fiber diameter, larger voids, lower junction density, and network coarsening, whereas x-PVA/HA showed a more moderate diameter increase together with local compaction and preservation of a more interconnected architecture. Importantly, the fibrous morphology was retained in all conditions, without network collapse or transition to a continuous film-like structure. These findings indicate that the ester-crosslinked PVA-based membranes remained morphologically stable under the investigated post-treatment conditions, while showing a clear formulation-dependent response to UV-C + simulated microgravity exposure.

The incubation conditions used in the present study, including a temperature of 37 °C and a humidified atmosphere, were selected to provide a controlled and identical environment for static and RPM-exposed samples. In crewed spacecraft, cabin temperature and humidity are actively controlled; current NASA human-system standards identify environmental conditions of approximately 20 °C–25 °C and 30%–60% relative humidity as supportive of crew comfort and performance (NASA, 2026). In this context, the present study provides a preliminary assessment of the short-term stability of the nanofiber mats under simulated microgravity conditions relevant to potential transport and storage scenarios.

### 3.2 Water vapor transmission properties of x-PVA and x-PVA/HA membranes

The water vapor transmission rate (WVTR) of the electrospun x-PVA and x-PVA/HA membranes was evaluated by a gravimetric cup method in order to assess their ability to regulate vapor exchange under conditions relevant to wound dressing applications. A schematic representation of the experimental setup and the corresponding WVTR values are shown in Figure 3. Both membrane formulations exhibited WVTR values markedly lower than the blank control, with statistically significant differences for both x-PVA and x-PVA/HA compared with the control (p < 0.0001; Figure 3b). x-PVA membranes showed a WVTR of 2,552.6 ± 66.7 g/m^2^·day, while x-PVA/HA membranes exhibited a comparable value of 2,492.6 ± 21.6 g/m^2^·day. No statistically significant difference was observed between the two membrane formulations. By contrast, the control system without a membrane showed a substantially higher WVTR of 5,693.9 ± 37.1 g/m^2^·day (Figure 3b).

**FIGURE 3:**
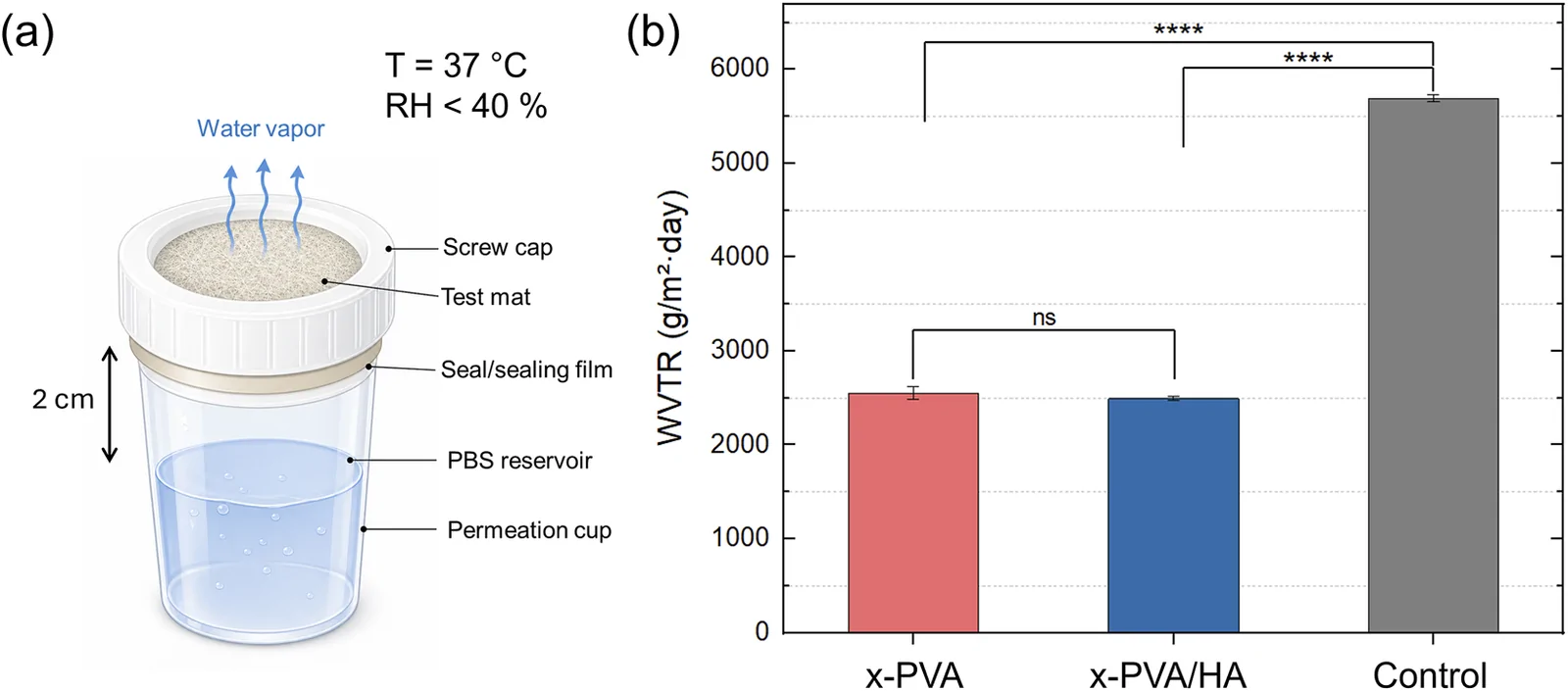
Water vapor transmission rate (WVTR) analysis of electrospun x-PVA and x-PVA/HA membranes. **(a)** Schematic representation of the gravimetric cup method used for WVTR measurement, showing the membrane fixed over a PBS reservoir and the testing conditions (37 °C, RH < 40%). **(b)** WVTR values of x-PVA, x-PVA/HA, and the blank control without membrane. Data are presented as mean ± SD (n = 3 independent measurements per condition). Statistical differences were evaluated by one-way ANOVA followed by Tukey’s multiple-comparison test. ****p < 0.0001; ns, not significant.

WVTR is a key parameter for wound dressing materials because it contributes to moisture regulation at the wound interface. Excessively low vapor transmission may favor exudate accumulation and maceration, whereas excessively high vapor transmission may promote wound dehydration. Previous studies reported that normal skin shows relatively low vapor loss, while wounded tissues may exhibit markedly higher values depending on wound type and exudation level (Svensby et al., 2024). In addition, controlled WVTR has been shown to support wound healing, with an optimal value of approximately 2028 g/m^2^·24 h reported for polyurethane membrane dressings in a wound healing model (Xu et al., 2016). These values place the x-PVA and x-PVA/HA membranes in an intermediate vapor-transport regime, in which water vapor loss is reduced compared with free evaporation but remains sufficiently high to support breathable dressing behavior. Both formulations reduced free water evaporation compared with the blank control while maintaining substantial vapor transport, suggesting that the electrospun mats acted as breathable barriers under the investigated conditions. Therefore, from the perspective of wound dressing design, both formulations showed breathable vapor-regulating behavior, with x-PVA/HA combining this property with the biological relevance of HA for wound-contact applications.

### 3.3 Infrared spectroscopic analysis of x-PVA and x-PVA/HA nanofiber mats

FTIR-ATR spectroscopy was performed to assess whether UV-C sterilization and subsequent exposure to simulated microgravity induced detectable chemical modifications in the electrospun x-PVA and x-PVA/HA nanofiber mats. The full spectra of the untreated and post-treated membranes are shown in Figure 4a, while magnifications of the C–H stretching region are reported in Figures 4b,c. Overall, all spectra retained the main vibrational features of the ester-crosslinked PVA-based systems, indicating that the chemical structure of the nanofibrous networks was preserved after UV-C treatment and 48 h incubation at 37 °C under either static or simulated microgravity conditions.

**FIGURE 4:**
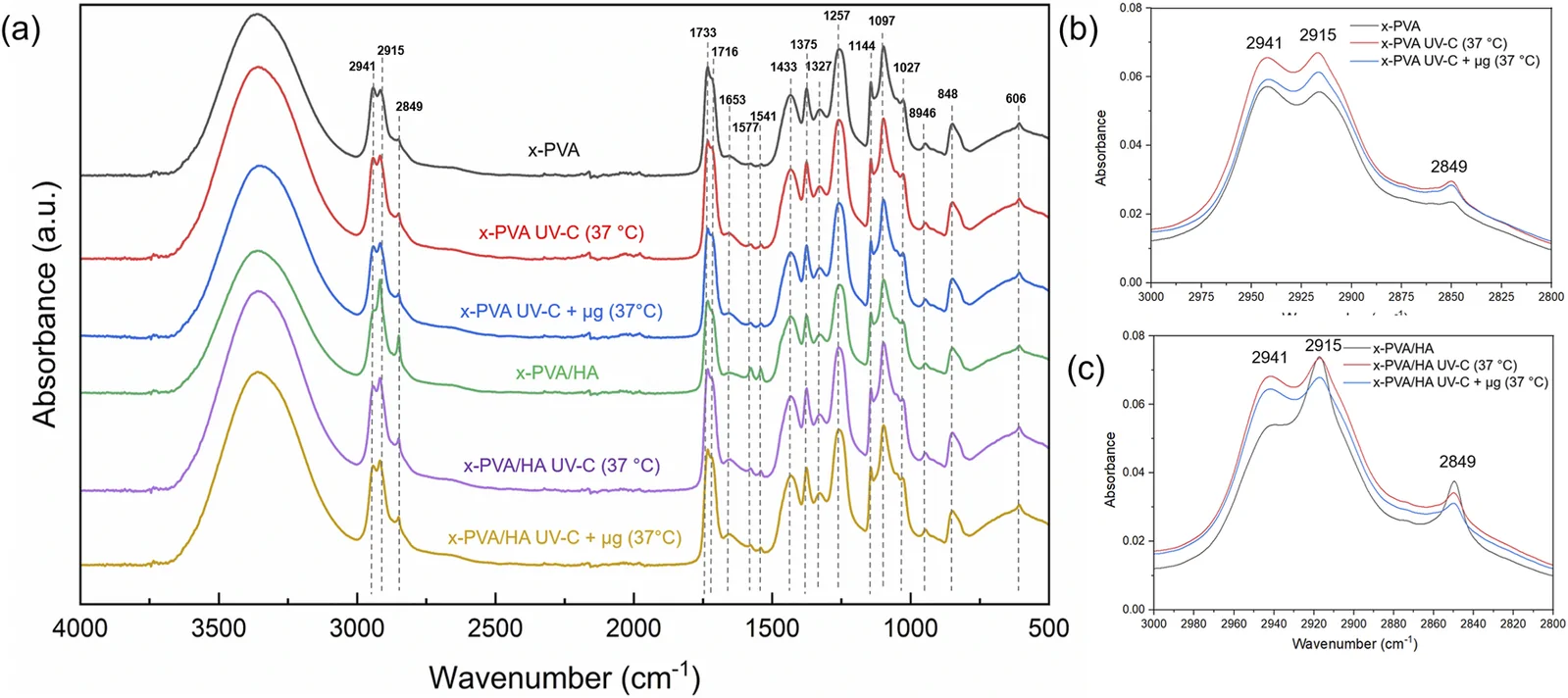
FTIR-ATR analysis of electrospun x-PVA and x-PVA/HA nanofiber mats before and after post-fabrication treatments. **(a)** Full FTIR-ATR spectra of untreated, UV-C-treated, and UV-C + μg-treated samples. Spectra are vertically shifted for clarity. **(b)** Magnification of the C–H stretching region between 3,000 and 2,800 cm^−1^ for x-PVA samples. **(c)** Magnification of the C–H stretching region between 3,000 and 2,800 cm^−1^ for x-PVA/HA samples. UV-C-treated samples were incubated at 37 °C for 48 h under either static conditions or simulated microgravity. Main absorption bands are indicated by dashed lines.

The broad absorption band in the 3,600–3,100 cm^−1^ region was assigned to O–H stretching vibrations of PVA and HA hydroxyl groups and to hydrogen-bonding interactions within the polymeric network. The bands at 2,941, 2,915, and 2,849 cm^-1^ were attributed to aliphatic C–H stretching vibrations of the PVA backbone. In the carbonyl region, the bands at 1,733 and 1,716 cm^−1^ were assigned to C=O stretching vibrations associated with ester carbonyl groups generated by citric-acid-mediated crosslinking, with possible contributions from residual carboxylic groups of CA and acetate-related groups in partially hydrolyzed PVA. These assignments are consistent with previous FTIR analysis of thermally crosslinked PVA/HA/CA electrospun nanofibers prepared using citric acid as crosslinking agent (Ciarleglio et al., 2026a). The signals observed around 1,653, 1,577, and 1,541 cm^−1^ were attributed to overlapping contributions from absorbed water, carboxylate groups, and, in x-PVA/HA, amide-related vibrations of hyaluronic acid, in agreement with reported IR band assignments for citric-acid-crosslinked PVA/HA nanofibers and hyaluronan-based systems (Ciarleglio et al., 2026a; Haxaire et al., 2003). The bands at 1,433 and 1,375 cm^−1^ were assigned to CH_2_ bending/symmetric carboxylate vibrations and C–H wagging/bending modes of PVA, respectively.

In the fingerprint region, the bands at 1,327, 1,257, 1,144, 1,097, and 1,027 cm^−1^ were mainly related to C–H/O–H bending and C–O/C–O–C stretching vibrations of the crosslinked PVA-based network. In particular, the band around 1,144 cm^−1^ was considered within the C–O–C/C–O stretching region of the crosslinked PVA-based network. Additional lower-wavenumber bands, including those around 946, 894, 848, and 606 cm^−1^, were attributed to skeletal vibrations and C–C/C–O modes. No new intense absorption bands appeared after UV-C treatment or after UV-C treatment followed by simulated microgravity exposure. This suggests that the applied post-treatments did not produce marked chemical degradation or extensive formation of new FTIR-detectable chemical species. The main spectral features of the x-PVA and x-PVA/HA networks were therefore preserved under the investigated conditions.

Treatment-dependent changes were mainly observed in the C–H stretching region. To describe these variations, the I_2941_/I_2915_ peak-height ratio was calculated from baseline-corrected spectra as an internal semi-quantitative descriptor of the C–H stretching envelope. In x-PVA membranes, this ratio changed only slightly, from 1.03 in the untreated sample to 0.97 after UV-C treatment and 0.98 after UV-C + μg exposure. In x-PVA/HA membranes, the ratio increased from 0.73 to 0.87 and 1.01 after UV-C and UV-C + μg exposure, respectively, indicating a more evident treatment-dependent modification of the C–H stretching envelope in the HA-containing formulation. Together with the preservation of the main carbonyl and fingerprint-region bands, these intensity variations are consistent with local changes in polymer-chain environment and network organization, particularly in x-PVA/HA, without FTIR-ATR evidence of marked degradation.

### 3.4 Thermal properties of x-PVA and x-PVA/HA nanofiber mats

DSC analysis was carried out to investigate the influence of UV-C sterilization and simulated microgravity exposure on the thermal behavior of the electrospun x-PVA and x-PVA/HA nanofiber mats. Representative thermograms in the glass transition and melting regions are shown in Figures 5a,c, while the corresponding T_g_ and T_m_ values are reported in Figures 5b,d. Both formulations exhibited a treatment-dependent modification of their thermal transitions. In the x-PVA system, the untreated membrane showed a T_g_ of 48.78 °C ± 1.86 °C and a T_m_ of 186.48 °C ± 0.81 °C. After UV-C treatment and static incubation at 37 °C, T_g_ decreased to 43.36 °C ± 0.89 °C, while T_m_ shifted to 173.31 °C ± 1.07 °C. Following UV-C treatment and simulated microgravity exposure, T_g_ and T_m_ were 46.54 °C ± 1.18 °C and 175.18 °C ± 1.37 °C, respectively. This indicates that the major thermal shift occurred after UV-C exposure and incubation at 37 °C, whereas the additional simulated microgravity step did not further depress the main transition temperatures.

**FIGURE 5:**
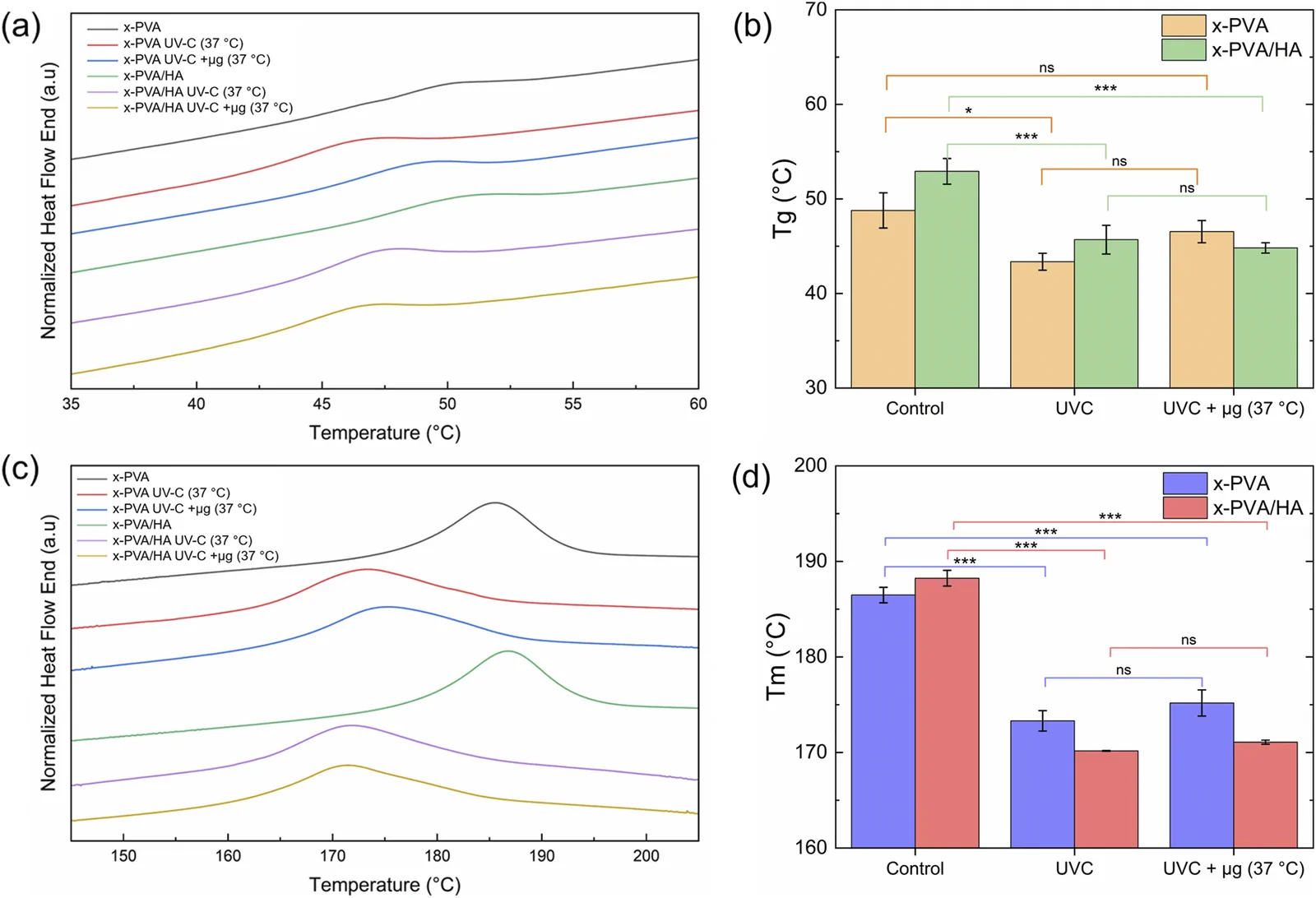
Thermal behavior of electrospun x-PVA and x-PVA/HA nanofiber mats before and after post-fabrication treatments. **(a)** Representative DSC thermograms in the glass-transition region and **(b)** corresponding Tg values. **(c)** Representative DSC thermograms in the melting region and **(d)** corresponding Tm values. UV-C-treated samples were incubated for 48 h at 37 °C under either static conditions or simulated microgravity. Data in panels **(b,d)** are presented as mean ± SD from three independent specimens per experimental condition (n = 3). Statistical differences were evaluated by two-way ANOVA followed by Tukey’s multiple-comparison test. *p < 0.05, **p < 0.01, ***p < 0.001; ns, not significant.

A comparable behavior was observed for the x-PVA/HA membranes. The untreated sample displayed a T_g_ of 52.92 °C ± 1.36 °C and a T_m_ of 188.24 °C ± 0.82 °C. After UV-C treatment under static conditions, these values decreased to 45.69 °C ± 1.52 °C and 170.16 °C ± 0.04 °C, respectively. The UV-C + μg-treated samples showed closely comparable transition temperatures, with T_g_ and T_m_ values of 44.82 °C ± 0.52 °C and 171.08 °C ± 0.21 °C. Therefore, also in the HA-containing system, simulated microgravity exposure did not induce an additional pronounced thermal shift with respect to the UV-C-treated static control.

The decrease in T_g_ and T_m_ observed after UV-C treatment followed by incubation at 37 °C may reflect treatment-induced rearrangements of the ester-crosslinked PVA-based network, including changes in chain mobility, hydrogen-bonding interactions, and the organization of ordered domains. This interpretation is consistent with previous studies reporting that UV irradiation can affect the molecular organization of PVA-based materials (Kaczmarek and Podgórski, 2007), and that UV exposure at 254 nm may modify the crystallinity-related thermal behavior of PVA-containing biomaterial blends (Sionkowska et al., 2009). In the present membranes, the downward shift of T_g_ and T_m_ is therefore consistent with moderate photochemical/structural rearrangement, involving increased chain mobility, changes in hydrogen-bonding interactions, and reorganization of ordered PVA domains. In hydrophilic PVA-based systems, these transitions are strongly affected by local water–polymer interactions and by the balance between amorphous and ordered domains. The absence of new intense degradation bands in the FTIR-ATR spectra suggests that these thermal changes were not associated with extensive chemical degradation, but rather with rearrangements detectable by DSC. The melting enthalpy showed a formulation-dependent response. In x-PVA membranes, ΔH_m_ remained essentially unchanged after UV-C treatment and decreased after UV-C + μg exposure, from 36.88 ± 1.58 J/g in the untreated sample to 31.69 ± 1.58 J/g. Conversely, x-PVA/HA membranes exhibited an increase in ΔHm after treatment, reaching 40.90 ± 2.68 J/g after UV-C exposure and 57.46 ± 3.47 J/g after UV-C + μg exposure. This behavior suggests that HA influenced the structural response of the crosslinked network, possibly favoring local chain packing or partial reorganization of ordered domains during incubation at 37 °C. However, thermal events in hydrated or partially hydrated PVA-based matrices may include overlapping contributions from residual water, polymer relaxation, hydrogen-bond rearrangement, and crystalline reorganization. Therefore, ΔH_m_ was used as a comparative thermal descriptor rather than as an absolute crystallinity index. Statistical analysis confirmed treatment-dependent differences in Tg and Tm, while no significant additional difference was observed between the UV-C-treated static and UV-C + μg conditions within either formulation for the main thermal transitions (Figures 5b,d).

Overall, DSC results indicate that UV-C treatment followed by incubation at 37 °C altered the thermal behavior of both x-PVA and x-PVA/HA nanofiber mats, mainly by shifting T_g_ and Tm toward lower temperatures. In contrast, simulated microgravity exposure did not produce a substantial additional change in the main thermal transitions compared with the corresponding static controls. Combined with the FTIR-ATR results, these findings suggest that the post-fabrication treatments primarily affected polymer chain mobility, hydrogen-bonding organization, and microstructural rearrangement, while preserving the overall integrity of the ester-crosslinked PVA-based networks.

### 3.5 *In vitro* cytocompatibility of x-PVA/HA nanofiber membranes

The biological response to the sterilized x-PVA/HA nanofiber membranes was evaluated using three human cell lines with distinct phenotypic characteristics: NT2D1 human embryonal carcinoma cells, HEK293 human embryonic kidney cells, and HaCaT human keratinocytes. This experimental design was selected to assess cell viability, morphology, adhesion behavior, and membrane–cell interactions using complementary optical and fluorescence-based approaches.

HaCaT cells were included as the application-relevant keratinocyte model (Colombo et al., 2017; Deyrieux and Wilson, 2007; Scharffetter-Kochanek et al., 1992). NT2D1 and HEK293 cells were not intended to reproduce the cellular composition of the wound bed, but were used as complementary human cell systems providing distinct cytocompatibility readouts. Specifically, NT2D1 cells were used to examine cell morphology, cytoskeletal organization, and adhesion recovery (Corano Scheri et al., 2018; Leonetti et al., 2019; Gesualdi et al., 2020), whereas GFP-transfected HEK293 cells enabled fluorescence-based assessment of membrane integrity and cell viability, taking advantage of the high transfectability of this cell line (Thomas and Smart, 2005; Lin et al., 2015). Thus, the multi-cell approach was used to assess general cytocompatibility rather than to reproduce wound healing *in vitro*.

To test the effect of the x-PVA/HA on cell viability and morphological appearance, we used fluorescent markers to make the cells observable under the microscope. Rhodamine phalloidin was used to label the cytoskeletal actin of NT2D1 cells, GFP and PKH67 were used to make renal epithelial cells and human keratinocytes visible. PI exclusion assays were used to assess membrane integrity and cell death.

NT2D1 cells were included as a non-seminoma cell model previously used by our group to investigate cell behavior, signaling pathways, and malignant phenotype regulation (Corano Scheri et al., 2018; Leonetti et al., 2019; Gesualdi et al., 2020; Gesualdi et al., 2023). In addition, NT2D1-derived systems have been widely used in studies on cellular differentiation, neurogenesis, and pluripotent stem cell-like properties (Andrews, 1984; Goodfellow et al., 2011; Borlongan et al., 2025; Kedracka-Krok et al., 2025). NT2D1 cells seeded on the x-PVA/HA membrane surface initially deposited onto the material but failed to establish stable adhesion, maintaining a rounded morphology without evident spreading (Figure 6a). After 24 h, cells were mainly observed on the underlying plastic substrate of the Ibidi chamber, where they adhered successfully. Rhodamine-phalloidin staining revealed actin cytoskeleton organization comparable to that observed in control cells (Figures 6b,c). After 24 h of exposure to x-PVA/HA, NT2D1 cells were detached by trypsinization and reseeded on uncoated plastic to evaluate adhesion recovery. After 72 h, these cells showed complete adhesion recovery, with morphology comparable to that of control cells (Figures 6d,e). These results indicate that x-PVA/HA did not compromise NT2D1 cell survival or adhesive capacity.

**FIGURE 6:**
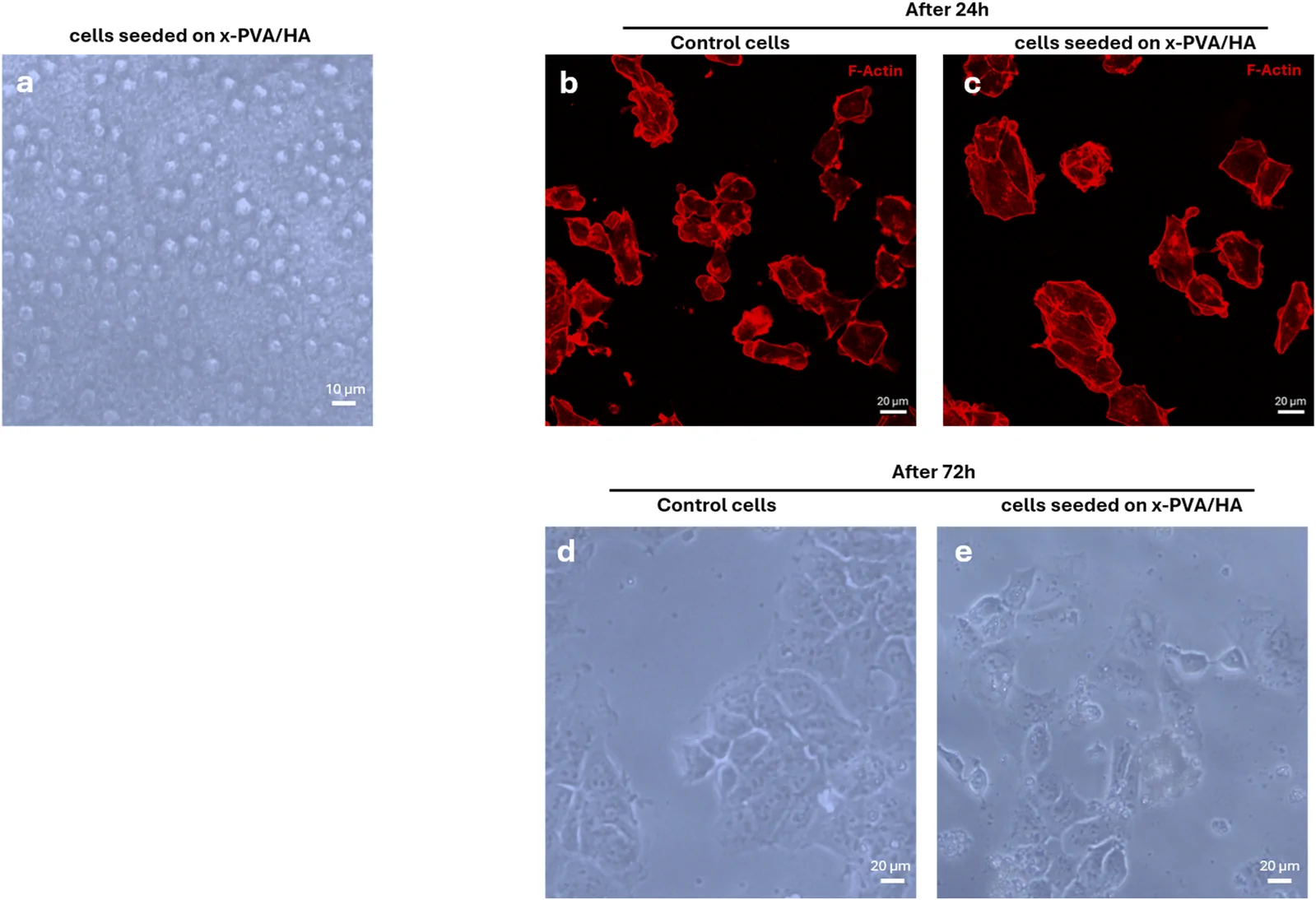
Effect of x-PVA/HA on NT2D1 Cell Viability and Morphological Appearance. **(a)** Representative optical microscopy images of NT2D1 cells seeded on x-PVA/HA (10× objective; scale bar = 10 μm). **(b)** Representative confocal microscopy images showing F-actin organization (red fluorescence) in control NT2D1 cells and **(c)** after 24 h of exposure to x-PVA/HA (20× objective; scale bar = 20 μm). **(d)** Representative images of NT2D1 cells reseeded after trypsinization and cultured for 72 h under control conditions and **(e)** on x-PVA/HA (20× objective; scale bar = 20 μm).

HEK293 cells were selected as an immortalized epithelial-like cell line widely used for their robust growth, high transfectability, and reliability in recombinant protein expression, vector production, and signaling studies (Thomas and Smart, 2005; Lin et al., 2015; Tan et al., 2021). In this work, HEK293 cells were transfected with GFP to allow fluorescence-based visualization, with a transfection efficiency higher than 80%. In control conditions, GFP-positive HEK293 cells adhered to the plastic substrate. In the presence of x-PVA/HA, cells failed to adhere stably to the material and were observed as both plastic-adherent cells and suspended cells (Figure 7a). Cell death was evaluated by propidium iodide (PI) exclusion assay, with total cells identified by GFP fluorescence and dead cells identified as PI-positive cells. Quantitative analysis showed comparable percentages of PI-positive cells among the different conditions: 7.17% ± 0.17% in control cells, 5.18% ± 1.25% in x-PVA/HA-exposed adherent cells, and 7.4% ± 1.35% in x-PVA/HA-exposed suspended cells (Figure 7b). These results indicate that x-PVA/HA did not compromise HEK293 cell survival.

**FIGURE 7:**
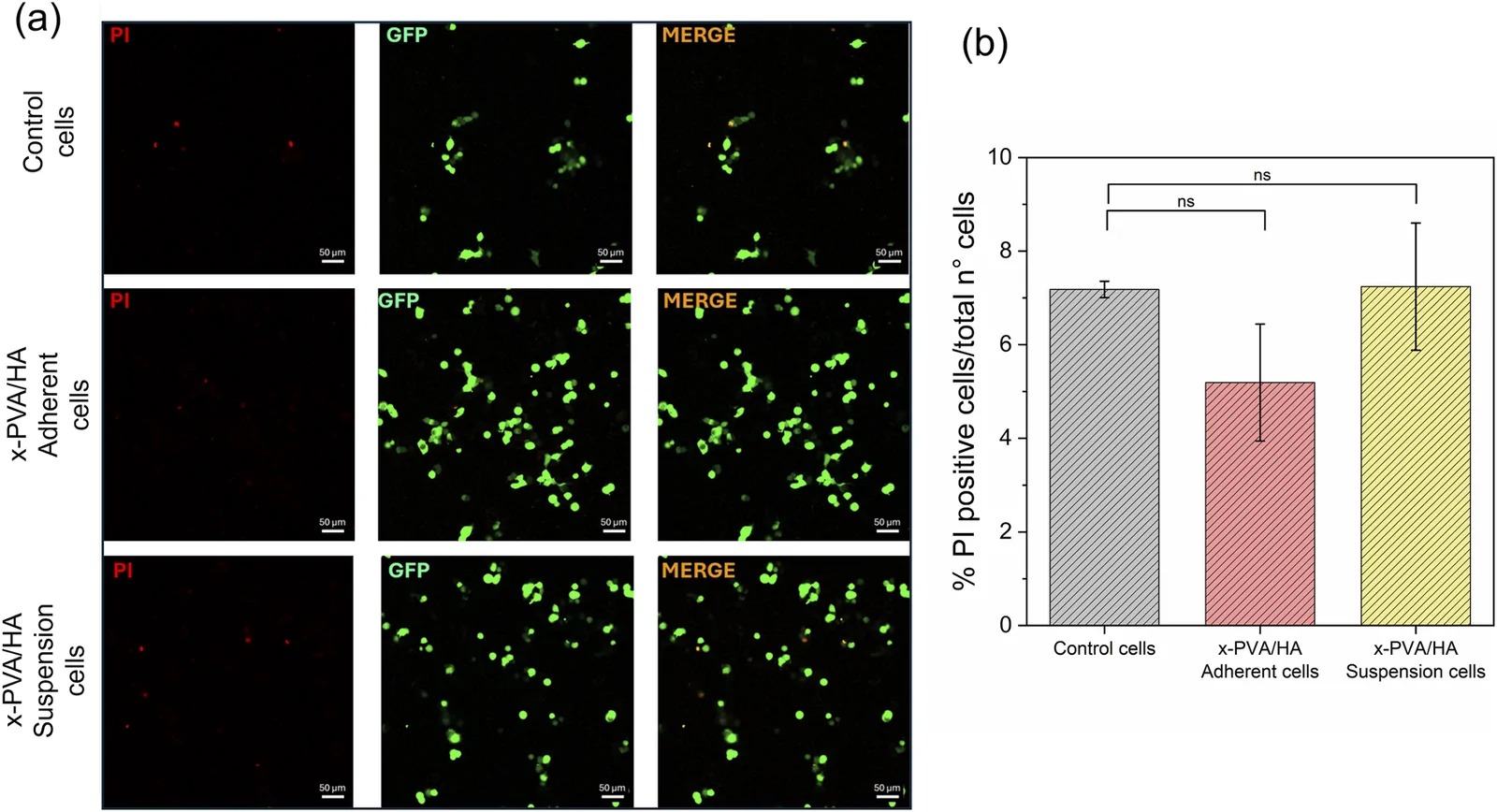
Effect of x-PVA/HA on HEK293 Cell Viability. **(a)** Confocal microscopy orthogonal projection images showing PI-positive cells (red fluorescence), GFP-positive cells (green fluorescence), and merged images under control, x-PVA/HA-adherent, and x-PVA/HA-suspension conditions after 24 h of exposure (20× objective; scale bar = 50 μm). **(b)** Percentage of PI-positive cells under control, adherent, and suspension conditions. Data are presented as mean ± SEM. No statistically significant differences were observed between control and x-PVA/HA-exposed conditions (Student’s t-test, p > 0.05; ns, not significant).

HaCaT cell lines were selected as immortalized, non-tumorigenic human keratinocytes that retain relevant features of epidermal differentiation and are commonly used as an *in vitro* model for keratinocyte migration, skin repair, and wound-healing-related studies (Colombo et al., 2017; Deyrieux and Wilson, 2007; Scharffetter-Kochanek et al., 1992). Their relevance is further supported by studies investigating microgravity-related effects on keratinocytes, fibroblast–keratinocyte interactions, and cell-adhesion remodeling in skin-associated models (Fedeli et al., 2022; Ricci et al., 2021). In this work, HaCaT cells were labeled with PKH67 Green Fluorescent Cell Linker to visualize the total cell population. PKH67-labeled HaCaT cells exposed to x-PVA/HA failed to establish stable adhesion to the material. In control conditions, cells adhered to the plastic substrate, whereas in the x-PVA/HA condition, two distinct populations were observed: plastic-adherent cells and cell aggregates displaying a spheroid-like three-dimensional morphology (Figure 8a). The coexistence of these two populations may reflect the heterogeneous adhesive environment generated by the presence of the membrane within the culture well. Cells reaching the exposed tissue-culture plastic could establish substrate adhesion and spread, whereas cells remaining above or in direct contact with the x-PVA/HA membrane had limited access to a permissive adhesive surface and therefore tended to maintain cell–cell interactions and form spheroid-like aggregates. This interpretation is consistent with previous observations of cell aggregation under reduced-adhesion culture conditions (Berardini et al., 2023; Su et al., 2013) and with the limited stable adhesion observed on x-PVA/HA under the investigated conditions. Cell death was evaluated on confocal microscopy series as the ratio between the sum intensity of PI-positive red fluorescence and the sum intensity of PKH67-positive green fluorescence. This fluorescence-intensity-based approach was used considering the three-dimensional organization of the HaCaT aggregates and is consistent with integrated fluorescence analyses previously applied to multicellular spheroid models (Berardini et al., 2023). No significant difference was observed between control cells and x-PVA/HA-exposed adherent cells, with values of 8.540 ± 1.43 and 7.560 ± 0.67, respectively (p = ns; Figure 8b). The spheroid-like cell population also showed a low number of PI-positive cells, although it could not be directly compared with the control condition because spheroid-like aggregates were not formed in the absence of the membrane. These results confirm that contact with x-PVA/HA did not compromise HaCaT cell survival.

**FIGURE 8:**
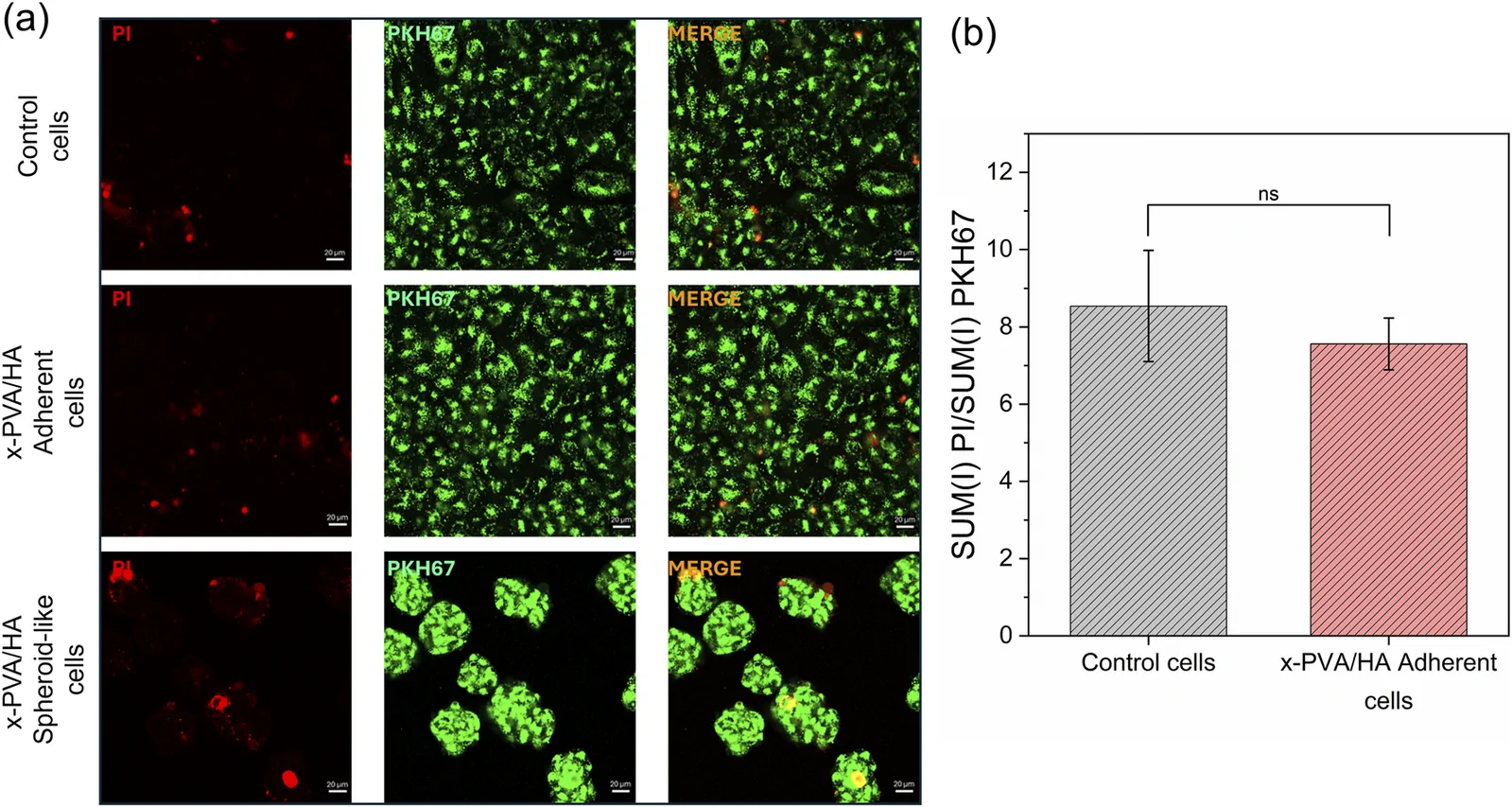
Effect of x-PVA/HA on HaCaT Cell Viability. **(a)** Confocal microscopy orthogonal projection images showing dead cells (PI^+^, red fluorescence), total cells (PKH67, green fluorescence), and merged images (PI^+^/PKH67) under control, adherent, and spheroid-like conditions after 24 h of exposure to x-PVA/HA (20× objective; scale bar = 20 μm). **(b)** Graphical representation of the ratio between the sum of red fluorescence intensity (PI^+^ dead cells) and the sum of green fluorescence intensity (PKH67, total cells) in control (8.54 ± 1.43) and x-PVA/HA-exposed adherent cells (7.56 ± 0.67). Data are presented as mean ± SEM. No statistically significant difference in the proportion of dead cells was observed between the two conditions (Student’s t-test: control vs. x-PVA/HA adherent cells, p > 0.05; ns, not significant). x-PVA/HA-exposed spheroid-like cells showed a low number of PI^+^ dead cells; however, these values were not quantitatively compared with the control condition because control cells cultured on plastic did not form spheroid-like structures.

Overall, the results obtained using these complementary experimental approaches show that NT2D1, HEK293, and HaCaT cells did not establish stable adhesive contacts with x-PVA/HA. Nevertheless, exposure to x-PVA/HA did not adversely affect cell viability, cytoskeletal organization, or the capacity of cells to recover adhesion over the investigated time frame. These findings indicate that UV-C-treated x-PVA/HA behaves as a cytocompatible and non-cytotoxic interface under the *in vitro* conditions investigated. Taken together, the use of three complementary assays across three human cell lines provided convergent and independent evidence of cytocompatibility, based on adhesion and cytoskeletal recovery as well as live/dead imaging. Although x-PVA/HA did not function as a cell-adhesive scaffold, it did not compromise cell survival or the ability of cells to recover on permissive substrates. These findings support its potential suitability as a protective wound-contact material, rather than as a cell-instructive scaffold designed to promote tissue regeneration.

## 4 Conclusion

This study investigated the response of ester-crosslinked x-PVA and x-PVA/HA electrospun nanofiber mats to UV-C sterilization followed by short-term incubation under static or simulated microgravity conditions. Both formulations preserved the continuous fibrous architecture after treatment, although SEM analysis revealed treatment-dependent morphological changes. UV-C + simulated microgravity exposure was associated with an increase in mean fiber diameter and local remodeling of the fibrous network, more pronounced for x-PVA than for x-PVA/HA. These results indicate a formulation-dependent morphological response of the ester-crosslinked PVA-based membranes to the post-treatment environment.

x-PVA and x-PVA/HA membranes showed comparable WVTR values, lower than the blank control, indicating that both formulations acted as breathable vapor-regulating barriers under the investigated conditions. FTIR-ATR analysis showed that the main absorption features of the ester-crosslinked PVA-based networks were retained after UV-C sterilization and simulated microgravity exposure, with no evidence of marked chemical degradation. DSC results indicated treatment-induced changes in thermal transitions, mainly after UV-C sterilization and incubation at 37 °C, while simulated microgravity did not produce a pronounced additional shift compared with the corresponding static controls. *In vitro* assays performed on the sterilized x-PVA/HA membranes showed that exposure to the material did not impair the viability of NT2D1, HEK293, or HaCaT cells. Although the membrane surface did not promote stable cell adhesion, exposed cells maintained cytoskeletal organization, low levels of cell death, and adhesion recovery on permissive substrates. These findings support the cytocompatibility of the x-PVA/HA nanofibrous membrane under the tested *in vitro* conditions.

Overall, the results indicate that citric-acid-crosslinked x-PVA/HA nanofibrous membranes combine breathable vapor-regulating behavior with preserved fibrous morphology, physicochemical stability and cytocompatibility after UV-C sterilization and short-term exposure to simulated microgravity-relevant conditions. These findings support further investigation of x-PVA/HA electrospun membranes as wound-contact materials for pre-sterilized, breathable, and cytocompatible applications in non-standard healthcare environments, including space-related scenarios, with future studies extending the present preliminary assessment to spacecraft-specific storage conditions and longer exposure periods.

## Data Availability

The raw data supporting the conclusions of this article will be made available by the authors, without undue reservation.
